# Renewed Avenues through Exercise Muscle Contractility and Inflammatory Status

**DOI:** 10.1100/2012/584205

**Published:** 2012-05-03

**Authors:** Nelo Eidy Zanchi, Felipe Natali Almeida, Fábio Santos Lira, José César Rosa Neto, Humberto Nicastro, Claudia Ribeiro da Luz, Mário Alves de Siqueira Filho, Vitor Felitti, Mariz Vainzof, Marilia Seelaender, Jacques R. Poortmans, Antonio Herbert Lancha

**Affiliations:** ^1^Laboratory of Applied Nutrition and Metabolism, Physical Education and Sport School, University of São Paulo, 05508-030 São Paulo, SP, Brazil; ^2^Cancer Metabolism Research Group, Institute of Biomedical Sciences, University of São Paulo, 05508-900 São Paulo, SP, Brazil; ^3^Human Genome Research Center, Institute of Biosciences, University of São Paulo, 05508-090 São Paulo, SP, Brazil; ^4^Department of Physiology and Biophysics, Institute of Biomedical Sciences, University of São Paulo, 05508-900 São Paulo, SP, Brazil; ^5^Department of Physiology, Division of Nutrition Physiology, Federal University of São Paulo, 04020-060 São Paulo, SP, Brazil; ^6^Laboratory for Applied Sport Nutrition, Faculty of Motility Sciences, Free University of Brussels, CP640 Brussels, Belgium

## Abstract

Physical inactivity leads to the accumulation of visceral fat and, consequently, to the activation of a network of inflammatory pathways which may promote development of insulin resistance, atherosclerosis, neurodegeneration, and tumour growth. These conditions belong to the “diseasome of physical inactivity”. In contrast, the protective effect of regular exercise against diseases associated with chronic inflammation may to some extent be ascribed to an anti-inflammatory effect. The so called “acute exercise threshold”, the complex mixture of several variables involved in exercise, such as type, volume, frequency, and intensity range is capable of inducing positive physiological adaptations and has been specifically addressed in the recent literature. The major concern is related to the level of the threshold: “exercise training shifts from a therapeutic adaptive intervention to one with potential pathological consequences”. Nonetheless, if the mechanical stimulus is too weak to disrupt cellular homeostasis, training adaptations will not occur. Answering these questions could present practical applications, especially during inflammatory diseases associated with detrimental muscle effects and could theoretically constitute a “new” therapeutic approach to treat/improve an inflammatory state. This paper aims to describe specific data from the literature regarding the effects of exercise on inflammatory diseases in order to promote a more sophisticated perspective on the anti-inflammatory effects of exercise.

## 1. Introduction

Daily physical exercise offers protection against many corporal disorders, which are responsible for numerous cases of mortality, including cardiovascular, metabolic, and neural diseases. First of all, we know that these disorders can coexist and share common mechanisms. In humans, type 2 diabetes is associated with impaired cognitive function (including learning, memory, and processing speed), accelerated cognitive decline and high risk to dementia and Alzheimer's disease [1]. In a 3-year followup, van Elderen et al. [2] observed that elderly patients with type 2 diabetes have accelerated progression of brain atrophy with significant consequences in cognition compared to subjects without type 2 diabetes, indicating that type 2 diabetes causes negative effects in neuronal integrity. Similarly, hypertension has been linked to presence of white matter abnormalities and Alzheimer's disease [3]. But in a study using an animal model of type 2 diabetes and hypertension, Yang et al. [3] showed that type 2 diabetes has a more pronounced effect on neurodegeneration than hypertension.

In addition, cancer patients with cachexia had higher mRNA expression of IL-6, TNF-R, and CRP levels [4], the same alterations occurred in obese patients. Other diseases present higher inflammatory markers. Duchene disease, is an example of a genetic disorder and is associated with the elevated presence of proinflammatory proteins [5].

In summary, inflammation is related to the pathogenesis of all of these diseases. Systemic low-grade inflammation is defined as two- to fourfold elevations in circulating levels of proinflammatory and anti-inflammatory cytokines, and chronic inflammation contributes to the development of atherosclerosis, insulin resistance, tumor growth, cancer, and neurodegeneration [6].

Many reports show the benefits of physical exercise improving type 2 diabetes [7], blood pressure [8], atherosclerosis and obesity [9], Alzheimer's disease and dementia [10], cognitive impairment [11], cancer [12], and other.

Even though the positive effects of regular physical exercise in all these disorders, some questions need to be clarified and have been discussed in this paper, for example “what is the link of all these diseases?” and “how can exercise influence these disorders?”. Thus, this paper focuses on describing specific data from the literature regarding the effects of exercise training on a wide range of inflammatory diseases to foster a more sophisticated perspective on exercise and inflammation.

## 2. What Is the Relationship between Exercise and Inflammation?

Physical inactivity leads to the accumulation of visceral fat and consequently to the activation of a network of inflammatory pathways that promote the development of insulin resistance, atherosclerosis, neurodegeneration, tumour growth, and other diseases associated with physical inactivity. According to Pedersen [13], these diseases belong to the “diseasome of physical inactivity”, a recent expression that describes the supra-cited effects of physical inactivity on the onset of chronic inflammation. This network of inflammatory pathways appears to be activated by environmental changes, which began with the introduction of agriculture and the domestication of animals about 10.000 years ago and occurred too recently to have triggered adaptations in the human genome [14, 15]. Consequently, the occurrence of chronic diseases, which manifest primarily in “modern civilizations,” is not due solely to genetic factors but rather to hypokinesia associated with impaired nutritional habits [16].

In contrast, the protective effect of regular exercise against diseases associated with chronic inflammation may to some extent be ascribed to a small inflammatory effect, which reinforces anti-inflammatory response. It was recently demonstrated by Pedersen's group that cytokines and other peptides are expressed and released by contracting muscle fibers and act to induce paracrine or endocrine effects [13]. Cytokines are a biologically active protein that holds several body functions and are known products of the immune system and inflammation [17]. In line with the term “adipokines” which covers cytokines and peptides produced and secreted by white adipose tissue (adipocytes), the term “myokines” is created and is used to describe cytokines and other peptides, which are produced and secreted by muscle fibers [13, 17].

For many years, researchers are interested to find a link between muscle fibers contraction and changes in several organs. The idea that signalling pathways from contracting muscles to other organs that were not only mediated by nervous system was supported by the findings from electrical stimulation of paralyzed muscles in patients with spinal cord injuries [18]. This research demonstrated the obvious: different compounds can be secreted by muscle fibers (i.e., muscle-derived humoral factors) and skeletal muscle should be classified as “endocrine organ” [13].

The first identified and most studied is the gp130 receptor cytokine interleukin-6 (IL-6). IL-6 is considered a myokine due to its increased release (up to 100-fold) in plasma during physical exercise and is dependent on exercise intensity, duration, the mass of muscle recruited, and endurance capacity [13, 17, 19]. Since the discovery that mechanical stimuli of skeletal muscle is capable of producing and secreting IL-6, this cytokine became well studied in relation to exercise training [20].

In summary, IL-6 mRNA is upregulated and its transcriptional rate is enhanced in contracting skeletal muscle. In addition, protein content of IL-6 and IL-6 released from skeletal muscle during exercise is increased [17]. IL-6 acts locally in muscle fibers through activation of AMPK and when released into the circulation, peripherally in several organs, mediates its effects by biding to its receptor, gp130 [6].

Importantly, IL-6 secreted during exercise exerts a strong inhibitory effect on the expression of several proinflammatory cytokines, including an increased anti-inflammatory interleukin-10 (IL-10) and decreased tumor necrosis factor alpha (TNF-*α*) expressions, which may be strictly linked to insulin resistance [21]. Thus, regular exercise seems to be an interesting *sine qua non *condition to avoid chronic diseases related to chronic inflammation in modern societies and its protective effects may be ascribed to an anti-inflammatory effect.

## 3. Exercise Threshold and Types

Chronic aerobic exercise training or resistance training might presumably induce the same degree of anti-inflammatory effects, whereby the degree of effect appears to be related to exercise variables and the volume of muscle mass involved in the mechanical stimuli [22]. Consequently, manipulations of exercise variables are vital to achieve positive or negative effects. The so called “acute exercise threshold” is defined as the complex mixture of several variables involved in exercise, such as exercise type, volume, and intensity range, capable of inducing positive physiological adaptations on remodelling. The major concern related to the level of threshold is: “exercise training shifts from a therapeutic adaptive intervention to one with potential pathological consequences” [23]. Nonetheless, if the exercise stimulus is too weak to disrupt cellular homeostasis (below this threshold), training adaptations may not occur. This information is extremely relevant because different inflammatory states might demand different types of exercise (i.e., resistance exercise or endurance exercise) as well as quantity (volume of training). This means that an inflammatory muscle state caused by mutations in proteins inside the muscle (i.e., Duchenne muscular distrophy) could demand distinct degree and type of exercise of an inflammatory state provoked by excess of visceral fat and insulin resistance. The differences in these responses may be explained by tissue-specific responses (i.e., adaptation in skeletal muscle and pathological and secondary in adipose tissue). For example, in Duchenne dystrophy skeletal muscle structure is impaired because of the absence of the protein dystrophin and an imbalanced mechanical stimuli may shift from a “therapeutic adaptive intervention to one with potential pathological consequences” [23]. In case of high adiposity, exercise will be intended to reduce visceral fat, decreasing proinflammatory and increasing anti-inflammatory proteins with more energy expenditure. Thus, the “acute exercise threshold” should be extremely individualised in according to the type of disease and exercise. However, in view of human genetic variability and exercise training patterns, a practical standard consensus to the mechanical stimuli to be applied in order to promote therapeutic effects under inflammatory states is not available. However, providing a bird's-eye view on what is known and unknown about the physiological and biochemical mechanisms involved in exercise-induced anti-inflammatory effects, the resulting map is surprisingly detailed in some areas such as obesity and cancer, whereas other areas such as Duchenne distrophy are still incompletely understood.

## 4. Would Exercise-Induced Chronic Effects Be Different than Acute?

According to Hawley et al. [24], although the major perturbations to cellular homeostasis and muscle substrate stores occur during exercise, the activation of several major cellular responses for chronic training adaptations takes place during the first few hours of recovery. These observations have contributed to the establishment of a paradigm which states that many chronic training adaptations are generated by the cumulative effects of the transient events that occur during exercise and recovery from each (acute) exercise bout [24]. In fact, the principles of exercise training assume that multiple sessions of acute exercise, imposed within an appropriate frame time, might stimulate and reinforce cellular and molecular processes that lead to a compensatory response [25]. Although increased mechanical stimulation-induced tension associated with resistance training has been shown to produce acute muscle damage (increasing TNF-*α* concentrations in muscle tissue) [26], repeated dynamic chronic resistance training may induce the known “repeated bout effect” which abolishes the acute muscle damage [27]. Therefore, it is expected that acute exercise responses should be completely different from those elicited by chronic exercise training [28]. For example, the attenuation of the acute phase inflammatory reaction is a known effect mediated by multiple sets of acute exercise performed on subsequent days [27], which means that acute and chronic responses are tightly linked, but the cellular responses generated after each occasion could be of a completely different nature. Corroborating these evidences, it was demonstrated that subjects submitted to 9 months of aerobic training showed reduced plasma concentration of C-reactive protein [29]. Furthermore, although increased mechanical stimulation-induced tension associated with resistance training has shown to produce acute muscle damage and increased TNF-*α* levels in muscle tissue [26], regular resistance training has demonstrated significant anti-inflammatory effects (e.g., decreases in TNF-*α* expression) both in aerobic exercised rats [21], strength trained rats [30], and in humans [31].

## 5. Models of Resistance Training and Therapeutic Purposes in Healthy Conditions

### 5.1. Models of Resistance Training for Healthy Adults

The importance of manipulating different resistance training variables resides in the unevenness of the biological response on muscle and nonmuscle cells that would be completely different depending on the stimulus. From this perspective, it is also known that such challenges to the exercised skeletal muscle increase several cellular adaptive processes, including the secretion of mechano- and insulin-like growth factors (MGF and IGF, resp.), which contribute to increased protein synthesis through activation of mammalian target of rapamycin (mTOR) pathway [32–34]. These factors also induce skeletal muscle damage and activate satellite cells through the former process, when chemoattractant substances (proinflammatory cytokines) are secreted by the muscle and inflammatory cells in order to attenuate the damage process and potentiate hypertrophy under physiologically healthy conditions [35, 36]. Thus, the discussion whether muscle damage is necessary to cause muscle hypertrophy seems significant. In physiological conditions, it could be a consequence of the choice of appropriate exercise variables ensuring the gain (or loss) of muscle mass under specific mechanical stress situations. However, if applied in other contexts (i.e., inflammatory diseases), such choices could be even more delicate and elicit impaired or increased responses in the signalling cascade involving skeletal muscle hypertrophy and the presence or absence of increased inflammation.

### 5.2. Specific Resistance Training: Therapeutic Purposes in Healthy Conditions

From the above considerations, the relevant questions rise as the following (1) Is there a necessity to cause muscle tissue damage under loading conditions to produce muscle hypertrophy? (2) Would such muscle hypertrophy in the absence of muscle damage be capable of inducing an anti-inflammatory milieu in the muscle tissue? (3) What is the lowest quantity of voluntary work capable to bring about such responses? Answering these questions could present practical applications, especially during inflammatory diseases associated with detrimental muscle effects and could theoretically constitute a “new” therapeutic approach to treated/improve this inflammatory state.

In order to answer the first and third questions, there is some speculation about the role of muscle damage in inducing hypertrophy. Some studies have demonstrated that exercise-induced muscle damage may not be a biological indicator of the hypertrophic response. Recently, our group studied trained rats using a previously described resistance training apparatus [37] during 12 weeks, employing a “therapeutic” resistance training protocol (i.e., resistance training protocol mainly composed of concentric forces, low frequency/low volume of training, twice per week, 8 repetitions per day, twice per day). High loads determined by the “volitional” percentage of maximum strength capacity (MVSC 80–95%) were used to avoid muscle damage. Our results showed that plasma phosphorylcreatine kinase was not modified after the 6th resistance exercise session measured during several moments (preexercise and 15 minutes, 24 hours and 48 hours after the 6th resistance training session). Moreover, at the end of the experiment (24 h after the last resistance training session), muscle histology samples of both groups (resistance-trained and control groups) were compared by eosin and hematoxylin staining methods, and no signs of inflammatory cells or muscle damage were visualised in either groups, nor were any centrally localised nuclei present in the muscle fibers (an indicator of muscle regeneration) [30]. In another study with the same model, muscle mass of resistance-trained animals increased by ~13%, with a significant decrease in the gene expression of atrogin-1 and MuRF-1 levels, the major muscle genes involved in the activation of genetic programs leading to a decrease in muscle mass through activation of the ubiquitin-proteasome system [38]. In contrast, a study by Flann et al. [39], which aimed to test the hypothesis that the symptomatic muscle damage is necessary to promote muscle remodeling in humans, demonstrated that levels of creatine kinase (CK), self-reporting of initial perceived soreness and exertion were significantly increased in the naive group compared to pretrained. However, muscle size and strength gains did not differ between groups and mean cross-sectional area and muscle strength showed a similar increase in both groups. These results suggest that remodeling and muscle hypertrophy can be initiated independently of any discernible damage to the muscle.

Regarding the second question, we studied rat muscles using the same resistance training protocol, but focused on the pro- and anti-inflammatory milieu. We did observe decreased protein expression of the proinflammatory cytokine TNF-*α* and IL-10/TNF-*α* ratio [40], regarded as an important indicator of the inflammatory status and disease-associated morbidity [21, 41, 42]. Thus, our results are compatible with the view of the “therapeutic” resistance exercise as a potential tool to promote anti-inflammatory activity in the muscle. Additionally, gene expression of TLR4 signalling, which stimulates inflammatory cytokine production [43], was also decreased by 60% in the plantaris muscle from trained rats, as compared to control rats. Molecular chaperones, such as heat shock protein 70 (Hsp70), have been found to increase under stressful conditions [44] but they were not increased in our resistance-trained animals when compared with the sedentary ones. In humans, Ogawa et al. [45] investigated the impact of 12 weeks of resistance exercise on inflammation in the elderly and showed significantly reduced circulating levels of C-reactive protein, Hsp70, and TNF-*α*. These proteins were significantly associated with muscle thickness. In conclusion, resistance training may assist in maintaining or improving muscle volume and reducing low-grade inflammation. Together, although in animal model, our results suggest the possibility of a “therapeutic” low frequency, low volume and nonperiodised exercise schedule, predominantly during a concentric resistance training protocol, as a way to achieve muscle hypertrophy and to improve the anti-inflammatory *milieu* in the absence of indirect markers of muscle damage.

## 6. The Obesity-Cachexia Paradox: Role of Exercise Training

Both cachexia and obesity may involve similar molecular defects since they represent the two extremes of muscle loss. In this context, exercise training seems to be a complementary therapeutic strategy which can positively affect both obesity and cachexia conditions.

 Several factors produced by the tumour and host tissues are suggested to play a part in the mediation of muscle loss in cachexia. These factors include proinflammatory cytokines such as TNF-*α*, IL-1*β*, and IL-6 and factors produced by tumour cells, each of which can be derived from the tumour itself and also from the host tissues [46]. Recently, several studies have characterised the relationship between chronic inflammation and the increase in inflammation markers, notably TNF-*α*, IL-1*β*, and IL-6, while transversal studies have shown a positive correlation between physical inactivity and low-grade systemic inflammation [13, 21, 47, 48], reinforcing the concept that a sedentary way of life is per se an inflammatory condition. A recent study by Sakurai et al. [49] indicated that exercise training induces antioxidant effects on visceral white adipose tissue (WAT) and that the levels of inflammation-related adipokines, such as TNF-*α* and monocyte chemotactic protein-1 (MCP-1) in WAT were lower in trained than in sedentary rats. These effects were more pronounced in visceral than in subcutaneous WAT.

 Eventually, frequent exercise training may abolish any kind of wasting in the skeletal muscle, and there appears to occur a cross-talk among skeletal muscles and adipose tissue such as occurances in cachexia induced by cancer or obesity, preventing exacerbated secretion and release of cytokines, mainly into adipose tissue. Recently, Zhou et al. [50] demonstrated a direct connection between increased concentrations of free fatty acids and increased rates of skeletal muscle proteolysis in C_2_C_12_ skeletal muscle cells. It is possible that this diminished proinflammatory cytokine production in skeletal muscle and WAT plus increased free fatty acids through exercise training could contribute to the normalisation of skeletal muscle proteolysis in obese and cachectic muscle. To reinforce such concept, sedentary rats presented increased TNF-*α* in the WAT tissue (specifically in the mesenteric tissue). In parallel, it was observed that IL-10 production almost is doubled in trained rats, changing the IL-10/TNF-*α* ratio in favour of anti-inflammatory properties [21]. Taken together, these results indicate that exercise training seems to be a comprehensive and low-cost alternative for the treatment of obesity-/cachexia-related chronic inflammatory diseases.

## 7. Mutations Leading to Myopathy: Role of Exercise Intensity

In Duchenne muscular dystrophy, mutations in the dystrophin gene lead to a deficiency of this protein in muscle sarcolemma, a component of the dystrophin-glycoprotein complex which forms an important link between the cytoskeleton and the extracellular matrix in the muscle. When a disruption of this link occurs due to a lack of dystrophin, a series of complicated events follow each other, resulting in muscle degeneration, significant weakness [51, 52], repeated cycles of degeneration-regeneration, progressive inflammation, and necrosis, with further destruction of the muscle fiber [53].

All of these observations have resulted in the suggestion that patients with Duchenne distrophy should not exercise [54, 55]. Moreover, intense exercise training seems to clearly induce muscle damage in mdx mice, an animal model for Duchenne distrophy [55, 56]. However, it is unknown if mdx mice are physiologically capable to adapt to exercise and what is the role of exercise intensity in these animals with increased ROS production. To elucidate these questions, Kaczor et al. [23] examined the effect of low-intensity training on markers of oxidative stress and observed that several markers of oxidative stress (malondialdehyde and protein carbonyls) were decreased especially in the fast-twitch muscles of mdx-trained mice when compared to the sedentary group. On the other hand, low-intensity training did not induce positive responses of the same oxidative stress markers in the wild-type animals. A possible explanation for this observation is related to the different exercise threshold presented by pathological *versus* nonpathological mdx mice. Thus, changing exercise intensity, it was decreased the oxidative stress markers in mdx mice challenging the generally accepted view that exercise is deleterious to skeletal muscle in the mdx model [23]. Nonetheless, it must be noted that the mdx mouse is a useful model to study the molecular changes induced by the absence of dystrophin, but clinical characteristics are manifested at a much lower intensity.

## 8. Conclusions and Perspectives

Exercise training is certainly an important, low-cost and effective therapy to treat several inflammatory diseases. However, most studies involving exercise training therapy are short-term, transversal studies, that is, the aforementioned results are of value but we do not know the impact of such adaptations on the long term. Thus, it is possible that appropriate adjustments to the training protocol may be an important factor to consider in future human studies.

 Regarding experimental research, a wide range of muscular and nonmuscular diseases are related to inflammation, but in some cases (Duchenne dystrophy for example), this relationship is a secondary alteration related to a primary defect in muscle proteins. In this case, should the effects of exercise efficacy be viewed in the same way as in other inflammatory diseases? Finally, the endoplasmic reticulum of secretory cells seems to be highly related with the induction of inflammation in several diseases such as diabetes and other diseases presenting a common denominator of chronic inflammation [57]. Thus, it is possible that the endoplasmic reticulum could be a next target to be evaluated under exercise conditions. More studies, especially those focused on protocol design and molecular responses, will help to shed light on the mechanisms involved in such responses.
